# Ending Preventable Child Deaths from Pneumonia and Diarrhoea in Afghanistan: An Analysis of Intervention Coverage Scenarios Using the Lives Saved Tool

**DOI:** 10.1155/2017/3120854

**Published:** 2017-02-19

**Authors:** Ariel Higgins-Steele, Khaksar Yousufi, Sharmina Sultana, Alawi Sayed Ali, Sherin Varkey

**Affiliations:** ^1^UNICEF Afghanistan Country Office, Kabul, Afghanistan; ^2^Afghanistan Ministry of Public Health, Child and Adolescent Health Directorate, Kabul, Afghanistan

## Abstract

*Background*. Despite improvements in child health, Afghanistan still has a heavy burden of deaths due to preventable causes: 17% of under-5 deaths are due to pneumonia and 12% are due to diarrhoea.* Objective*. This article describes the situation of childhood pneumonia and diarrhoea in Afghanistan, including efforts to prevent, protect, and treat the two diseases. It estimates lives saved by scaling up interventions.* Methods*. A secondary analysis of data was conducted and future scenarios were modelled to estimate lives saved by scaling up a package of interventions.* Results*. The analysis reveals that 10,795 additional child deaths could be averted with a moderate scale-up of interventions, decreasing the under-five mortality rate in Afghanistan from 55 per 1,000 live births in 2015 to 40 per 1,000 in 2020. In an ambitious scale-up scenario, an additional 15,096 lives could be saved. There would be a 71% reduction in child deaths due to these two causes between 2016 and 2020 in the ambitious scenario compared to 47% reduction in the moderate scenario.* Conclusion*. Significant reductions in child mortality can be achieved through scale-up of essential interventions to prevent and treat pneumonia and diarrhoea. Strengthened primary health care functions and multisector collaboration on child health are suggested.

## 1. Introduction

Afghanistan has made impressive gains in reducing maternal and child mortality in just over a decade. Child mortality has more than halved, decreasing to 55 deaths per 1,000 live births in 2015 [1] from 137 per 1,000 live births in 2000 [2]. Notwithstanding this progress, Afghanistan still has a heavy burden of child deaths due to preventable causes such as diarrhoea and pneumonia, which have overlapping risk factors. In 2015, 17% of all under-5 deaths were due to pneumonia and 12% due to diarrhoea [2]. There are approximately 94,000 deaths among children under five years every year in the country according to most recently available global estimates [2].

Afghanistan's Ministry of Public Health has made commitments to scale-up essential maternal, newborn, and child health interventions, including those that span the* Global Action Plan for Pneumonia and Diarrhea's *(GAPPD) areas of prevention, protection, and treatment [3]. Pneumococcal vaccine was introduced as part of routine immunization in 2013, and there are plans to introduce rotavirus vaccine in 2018. For treatment of diarrhoea, oral rehydration solution (ORS) is being scaled up and zinc is being introduced through codistribution of zinc-ORS packs across the country, starting in 2016. These initiatives are alongside ongoing efforts to improve coverage of routine vaccination, integrated management of childhood illness (IMCI), and child nutrition and water, sanitation, and hygiene (WASH) interventions.

This article describes the situation of childhood pneumonia and diarrhoea in Afghanistan, including efforts to prevent, protect, and treat the two diseases. Further, it estimates the potential additional child lives saved by scaling up the key related interventions in different scenarios, using the Lives Saved Tool (LiST).

## 2. Methods

This analysis is structured in line with the framework in the WHO and UNICEF's GAPPD (2013) and specifically the interventions in its three domains: prevent, protect, and treat [3].

### 2.1. Situation Analysis

A literature review was also conducted in PubMed and Google Scholar to identify existing data and evidence related to pneumonia and diarrhoea among children under five years of age, in terms of causes, prevention, protection, and treatment. Key words used were [“Afghanistan” and “pneumonia”], and [“Afghanistan” and “diarrhoea”]. Grey literature including policy and programme documents were captured by reviewing UNICEF Afghanistan's internal files as well as through Google searches. Articles and grey literature with relevant evidence on the situation/burden of these diseases and contributing factors, as well as public health responses to prevent, protect, or treat either illness, were retained from the last ten years.

### 2.2. Lives Saved Tool

The Lives Saved Tool (LiST), a software projection model, was used to estimate the number of deaths that can be averted by scaling up effective maternal and child health interventions. This software allows for simultaneous scaling up of interventions across the life cycle, from reproductive care through pregnancy and child birth to child health. The model and approach used in LiST are drawn from empirical evidence on effectiveness of individual interventions [4]. Description of uses of LiST and materials and methods used in the estimation of parameters are described in detail elsewhere [5, 6]. LiST has been used for planning purposes and to inform policy in low-resource settings [7], including model reductions due to pneumonia and diarrhoea [8–10].

Modelling a future scenario in LiST requires four sets of inputs: (i) intervention coverages that can be scaled up from baseline levels; (ii) measures of health status (e.g., levels of risk factors and population exposures and baseline cause-specific mortality estimates); (iii) estimates of intervention effectiveness; and (iv) target levels for interventions for a future year [4].

Data from the most recent national surveys were examined as part of establishing credible baseline data for the modelling exercise. These included the most recent Demographic and Health Survey (DHS) (2015) [1], WASH Joint Monitoring Programme (JMP) (2015) [11], National Nutrition Survey (NNS) (2013) [12], Multiple Indicator Cluster Survey (MICS) (2010) [13], and Emergency Obstetric and Newborn Care (EmONC) Needs Assessment (2010) [14].

Three scenarios were modelled for end line target coverage: a scenario with “moderate” coverage increases of selected interventions from 2016 to 2020, and a scenario with “ambitious” coverage increases from 2016 to 2020, and a scenario with coverage from 2016 to 2030 (see Table 1 for baseline and end line rates used in the models). Specialists in health, nutrition, and WASH were consulted for selecting baseline indicators for pneumonia and diarrhoea interventions when multiple sources were available, as well as in selecting target coverage levels for moderate and ambitious scale-up. Time periods modelled were 2016 to 2020 in line with Afghanistan's National Health Policy and Strategy and 2030 for a scenario aligned to the timeline set by the Sustainable Development Goals (SDGs).

One important methodological issue to acknowledge is that the LiST software first attributes impact to implemented preventive interventions (ordered sequentially from periconception, through pregnancy and delivery, followed by specific age groups) and then attributes impact to the available curative interventions, also within this sequential pattern. Therefore when both a preventive and a curative intervention are scaled up in the same scenario, the full effect of the change in coverage of the preventive intervention is calculated first then any residual deaths averted are calculated and attributed to the curative intervention [15].

In the scenarios, indicator levels were increased linearly to target levels in the future years. Some analyses using LiST select a predetermined target coverage level (e.g., 80%) for a majority of interventions and a higher target coverage (e.g., 90%) for those remaining, such as vitamin A supplementation and vaccines. Taking into account the current context in Afghanistan, for the ambitious scale-up scenario, we did not apply a blanket target level percentage. Instead, review of future targets took into account the current baseline level and recent trends for each indicator.

In our analysis, one indicator, rotavirus vaccine, was set at zero for 2016 and 2017, since its introduction is planned for 2018. All other interventions in LiST not directly associated with pneumonia and diarrhoea (skilled birth attendance, polio vaccine, etc.) were kept at their current levels for baseline and end line to isolate the effects of pneumonia and diarrhoea interventions.

## 3. Results

Pneumonia and diarrhoea are leading causes of under-five deaths in Afghanistan. Although the share of deaths in the neonatal period in Afghanistan is increasing overall for deaths among children under five years (representing approximately 40% of all under-five deaths), the burden of these diseases is much higher in the postneonatal period (contributing to approximately more than one in four of all deaths in the postneonatal period). In the case of neonatal deaths, only 2% of neonatal deaths are due to pneumonia and 0% due to diarrhoea.

**Figure 1:**
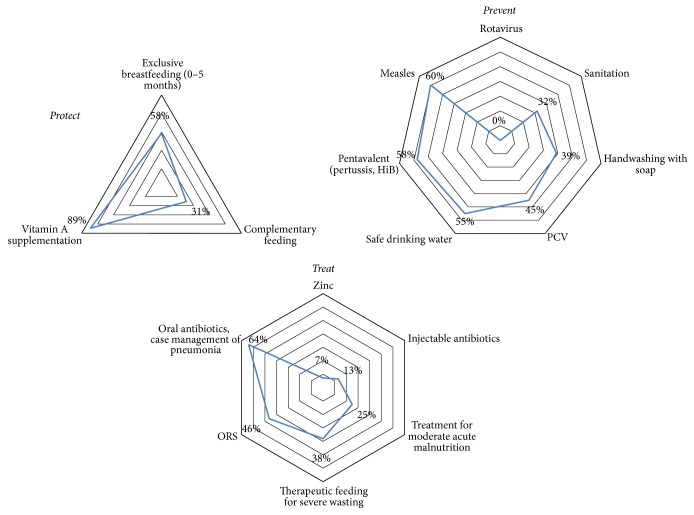
Baseline coverage levels in Afghanistan for interventions to protect, prevent, and treat pneumonia and diarrhoea, included in LiST.

Across baseline indicators on interventions that prevent, protect, and treat pneumonia and diarrhoea in Afghanistan, there are wide variations in coverage levels (Figure 1). Coverage of only one intervention—vitamin A supplementation—is high in Afghanistan; most intervention coverage rates are between 30% and 60%. Some interventions are currently at low levels, such as zinc for treatment of diarrhoea. Rotavirus vaccine has not yet been introduced in Afghanistan.

**Figure 2:**
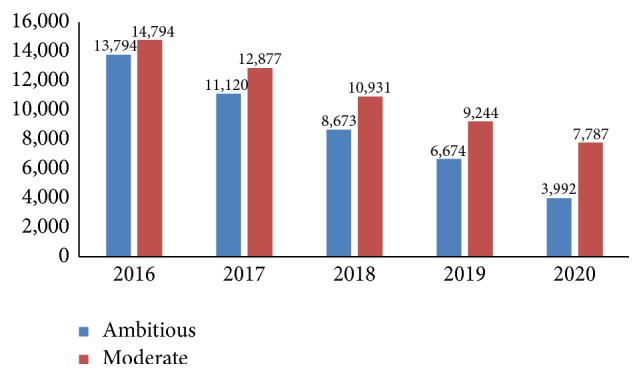
Number of pneumonia and diarrhoea deaths in moderate and ambitious scale-up scenarios (2016–2020).

The LiST analysis reveals that with moderate scale-up of pneumonia and diarrhoea interventions (Table 1), by 2020, a total of 10,795 additional child deaths caused by pneumonia or diarrhoea could be averted. In an ambitious scale-up scenario, an additional 15,096 lives could be saved by 2020. There would be an incremental decrease in under-five child deaths from pneumonia and diarrhoea in both scenarios, with a more rapid decline in an ambitious scale-up scenario of 71% reduction in child deaths due to these two causes between 2016 and 2020 (for diarrhoea, a 85% reduction; for pneumonia, a 63% reduction) compared to 47% reduction in the moderate scenario (for diarrhoea, a 35% reduction; for pneumonia, a 63% reduction) (Figure 2).

In these two scenarios, the majority of additional lives saved are in the postneonatal period, given that interventions are largely geared toward postneonatal prevention and treatment. In the moderate scenario, only 3.2% of additional lives saved (*n* = 1279) are in the neonatal period and in the ambitious scenario the figure is lower at 2.7% (*n* = 920).

In the moderate scale-up scenario, increases in pneumonia and diarrhoea interventions across the prevent, protect, and treat framework would result in decreasing the under-five mortality rate in Afghanistan from 55 per 1,000 live births in 2015 to 40 per 1,000 live births in 2020, keeping all other interventions related to maternal, newborn, and child health constant.

Further, in this scenario, pneumococcal vaccine saves the most additional lives (*n* = 2,239) of all interventions, followed by HiB in Pentavalent vaccine (*n* = 1,384) and promotion of breastfeeding (*n* = 1,153). Immunization provides a huge contribution to additional lives saved in this scenario, about 50% (*n* = 5,335) additional lives are saved by HiB, pneumococcal, DTP, and rotavirus vaccines combined. These results take into account the sequencing of intervention types in LiST modelling mentioned in Methods (Table 2).

**Figure 3:**
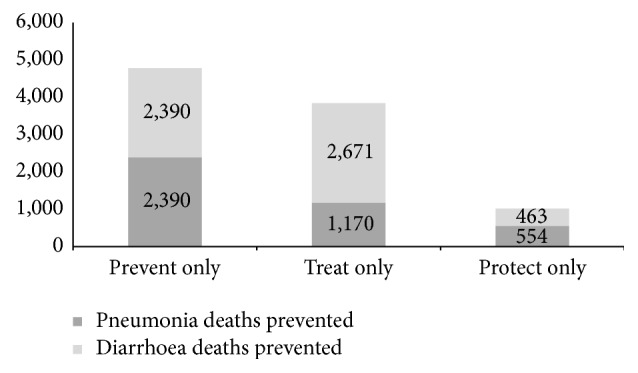
Additional deaths prevented in children under five years of age by cause (2016–2020).

We also modelled three different scenarios for 2020 that isolated each of the subgroups of interventions for protect, prevent, and treat with the moderate scale-up target figures. Predictably only scaling up a subset of interventions results in less additional lives saved overall. The prevent group of interventions (e.g., full routine vaccination, WASH) saved the most additional lives during the period (*n* = 4780), followed by treat (e.g., ORS, zinc, antibiotics, and therapeutic feeding; *n* = 3841 lives saved). Protect (e.g., breastfeeding, complementary feeding, and vitamin A supplementation) saved the least lives (*n* = 1170) (Figure 3) though it well established that healthy nutrition status contributes to less susceptibility to disease.

We also modelled a scenario for 2030 in line with the SDGs timeline. In a longer term, even with sustained high coverages of pneumonia and diarrhoea interventions, child mortality decreases to only 45 deaths per 1,000 live births, largely because as under-5 mortality will decline, neonatal causes will increase as a proportionate share of all causes and would represent 58% (*n* = 26,383) of all deaths in 2030 with all other interventions remaining constant in this model.

### 3.1. Limitations

While the effect size of individual interventions in LiST modelling is based on global evidence, the projections underestimate effects of nutrition and WASH interventions. Nutrition research has suggested that better modelling tools are needed to adequately capture the impact of nutrition on childhood mortality [16].

## 4. Discussion

We used LiST to simulate potential child lives saved in Afghanistan by scaling up interventions that help prevent, protect, and treat pneumonia and diarrhoea. The results show that moderate increases in coverage of essential interventions between 2016 and 2020 lead to reduction in overall number of child deaths by 10,795, or a reduction of 21 percentage points in child mortality (deaths per 1,000 live births) overall from 55 in 2016 to 40 in 2000.

Afghanistan's Basic Package of Health Services (BPHS), introduced more than a decade ago to quickly provide access to primary health care across the country after the fall of the Taliban government, improved national coverage of essential interventions. However, coverage levels of many indicators including those related to pneumonia and diarrhoea remain low [17, 18].

Predictably, the scenarios show that the full prevent, protect, and treat package is the most effective in saving the maximum number of additional child lives; though given low baseline coverage levels for some interventions, prevention and treatment interventions show comparatively larger results in terms of lives saved. Nutrition interventions require special attention, especially given high rates of malnutrition in Afghanistan—41% of children under five are moderately or severely stunted [12]. This is especially important since empirical evidence indicates that undernutrition (fetal growth restriction, suboptimum breastfeeding, stunting, wasting, and deficiencies of vitamin A and zinc) causes 45% of all deaths in children [19]. Likely the effects of poor nutrition among children in Afghanistan are underestimated. Attention to nutrition in the context of pneumonia and diarrhoea includes awareness-raising and interventions to improve infant and young child feeding after common illnesses, which is not widely practised in the country [20, 21] and region [22]. A recent study in one province in Afghanistan showed that children who had diarrhoea in the last two weeks were about two times more likely to be acutely malnourished than children with no such illness [23].

In the scenarios we have projected in this analysis, the reduction in pneumonia as a cause of death is at a comparatively slower rate than reduction of diarrhoea as a cause of death. This is largely due to the fact that more focus would be required on newborn pneumonia and sepsis for further reduction in pneumonia related deaths. Improvements in the care and treatment of newborns would require increases in institutional births, and recent estimates indicate that only half of women give birth in a health facility [1]. In addition, reducing newborn deaths due to pneumonia will require improvements in antenatal care and quality of case management, especially for newborns with severe and complicated infections [24].

Community health workers (CHWs), are considered “the world's most promising health workforce resource for enabling health systems in resource-constrained settings to reduce the burden of disease from serious, readily preventable or treatable conditions” [25]. In Afghanistan, due to gender dynamics that influence task allocation, CHWs work as female/male pairs with female CHWs accomplishing their maternal and child health related tasks with greater ease than male counterparts [26]. Ongoing capacity-building efforts for this cadre are needed, given the breadth of health promotion activities as well as high turnover rates due to the volunteer nature of the work in this country. Capacity building is also critical as zinc and ORS copacks for treatment of diarrhoea are being rolled out in 2016.

The important contribution of vaccination to the scenarios indicates that attention must be paid to improving vaccination coverages rates. At present and in the immediate future, there are huge opportunities to leverage resources invested in polio eradication in Afghanistan to strengthen routine vaccination and reach more children with full immunization coverage [27].

Studies have shown that care-seeking for childhood illness has improved in Afghanistan, but there remains scope for further improvement. For example, seeking health care by any provider for treatment of pneumonia among children aged 0–59 months increased significantly from 46% in 2003 to 77% in 2012, but with wide variation between groups and geographic locations [28].

Similarly, while primary care across the country has improved outpatient management of sick children in Afghanistan, the management of pneumonia and diarrhoea, especially, requires strengthening. This includes attention to overprescribing of antibiotics for diarrhoea cases [29]. Additionally, improvements in care must be complemented with strengthening of referral to higher level health facilities [30].

Finally, while WASH indicators are also better, more must be done in this area to decrease incidence of pneumonia and diarrhoea. A cohort study of children in Kabul with recurrent diarrhoeal illnesses found barriers to access and knowledge regarding the importance of handwashing and improved sanitation facilities, which already tends to be higher in urban areas [31]. Similarly, a randomized control trial on water and hygiene interventions in a rural province in Afghanistan found that multibarrier methods are required where there are several factors contributing to water contamination [32].

Ultimately, diarrhoea and pneumonia deaths among children are strongly associated with social determinants of health. A lack of or little education of mothers, child marriage, lack of or minimal maternal autonomy, shortage of basic material needs, and internal displacement have been shown to have independent and significant negative associations with child health and nutritional variables in Afghanistan [33].

## 5. Conclusion

Significant additional reductions in child mortality can be achieved through moderate scale-up of essential interventions to prevent and treat pneumonia and diarrhoea. Strengthened primary health care functions and multisector collaboration on child health are suggested. While the health facility approach is critical, it is not enough to achieve reductions in child mortality and morbidity due to these two diseases. Strengthening community awareness and demand for these interventions, as well as reinforcing healthy behaviors, are vital to make sustainable progress in reducing preventable child deaths due to pneumonia and diarrhoea.

## Figures and Tables

**Table 1:** Baseline and end line target coverage rates for interventions included in the Lives Saved Tool (LiST) modelled for scale-up scenarios.

Intervention	Baseline (most recent coverage data)	Source^*∗*^	Target 2020 moderate scale-up	Target 2020 ambitious scale-up	Target 2030 scale-up
Exclusive breastfeeding <1 month	74.5%	NNS 2013	84%	90%	90%
Exclusive breastfeeding 1 to 5 months	55.1%	NNS 2013	60%	70%	70%
Any breastfeeding 6 to 11 months	72.2%	NNS 2013	80%	90%	90%
Any breastfeeding 12 to 23 months	60.1%	NNS 2013	65%	75%	75%
Complementary feeding: education only	31.0%	NNS 2013	60%	90%	90%
Complementary feeding: supplementation and education	20.0%	NNS 2013	50%	70%	70%
Vitamin A supplementation	89.1%	NNS 2013	95%	95%	95%
Water connection in the home	12.0%	JMP 2015	20%	50%	50%
Improved sanitation (utilization of latrines or toilets)	32.0%	JMP 2015	50%	75%	75%
Handwashing with soap	39.0%	MICS 2010-11	50%	75%	75%
Hygienic disposal of children's stools	45.8%	MICS 2010-11	60%	90%	90%
Pentavalent vaccine	57.7%	DHS 2015	90%	95%	95%
Pneumococcal vaccine	44.9%	DHS 2015	90%	95%	95%
Rotavirus vaccine	0%	*∗∗*	90%	95%	95%
Measles vaccine	60.4%	DHS 2015	90%	95%	95%
Injectable antibiotics	13.0%	EmONC 2010	20%	50%	50%
Oral rehydration solution (ORS)	46.2%	DHS 2015	75%	90%	90%
Antibiotics for treatment of dysentery	64.0%	EmONC 2010	75%	90%	90%
Zinc for treatment of diarrhoea	7.1%	DHS 2015	60%	90%	90%
Oral antibiotics: case management of pneumonia	64.0%	MICS 2010-11	75%	90%	90%
Therapeutic feeding for severe wasting	38.0%	BNA 2015	60%	90%	90%
Treatment for moderate acute malnutrition	25.0%	BNA 2015	40%	80%	80%

^*∗*^See text for description of acronyms.

^*∗∗*^Planning to introduce it in 2018 in routine EPI schedule.

**Table 2:** Additional deaths prevented in children under five years of age by intervention by year.

Intervention	2016	2017	2018	2019	2020
Pneumococcal vaccine	0	576	1,139	1,693	2,239
*H. influenzae* b vaccine	0	359	707	1,049	1,384
Promotion of breastfeeding	0	305	596	879	1,153
Oral rehydration solution	0	448	752	959	1,086
Measles vaccine	0	206	406	601	792
Rotavirus vaccine	0	0	242	462	663
Oral antibiotics for case management of pneumonia in children	0	193	345	461	544
Therapeutic feeding for severe wasting	0	176	316	428	515
Improved sanitation (utilization of latrines or toilets)	0	106	201	287	366
Handwashing with soap	0	94	178	255	325
Appropriate complementary feeding	0	75	152	230	307
Water connection in the home	0	86	162	232	295
Zinc for treatment of diarrhoea	0	118	196	247	276
DPT vaccine	0	64	128	192	257
Hygienic disposal of children's stools	0	45	86	123	156
Improved water source	0	36	68	98	125
Vitamin A supplementation	0	23	43	61	78
Treatment for moderate acute malnutrition	0	24	43	56	65
Vitamin A for treatment of measles	0	28	46	55	56
Injectable antibiotics	0	15	29	42	55
Oral antibiotics	0	9	18	27	35
Antibiotics for treatment of dysentery	0	11	18	23	25
